# Using artificial intelligence as a technological tool in gynecologic and obstetric health: A narrative literature review

**DOI:** 10.1002/ijgo.70455

**Published:** 2025-08-18

**Authors:** Gustavo Gonçalves dos Santos

**Affiliations:** ^1^ Universidade de Ribeirão Preto, Campus Guarujá (UNAERP) São Paulo São Paulo Brazil

**Keywords:** artificial intelligence, digital health, gynecology, maternal mortality, mortality, obstetrics, science, technology, women's health

## Abstract

Maternal mortality remains a critical global public health issue, particularly in low‐ and middle‐income settings where failures in surveillance, early diagnosis, and clinical decision making compromise obstetric care. In this context, the present study aimed to critically review the scientific literature on the use of artificial intelligence (AI) in gynecologic and obstetric health, focusing on the prevention of avoidable deaths and severe maternal events. This is a narrative literature review with a qualitative and exploratory‐interpretative approach, conducted between May and June 2025 in relevant electronic databases, following structured axes for narrative reviews and well‐defined eligibility criteria. A total of 403 records were identified, of which 17 studies met the criteria and were included in the analysis, supported by the Interface de R pour les Analyses Multidimensionnelles de Textes et de Questionnaires software. The results showed that AI has been used to predict obstetric risks such as pre‐eclampsia, postpartum hemorrhage, and preterm birth through machine learning algorithms, neural networks and predictive models based on electronic health records and laboratory tests. Tools such as clinical decision support systems, portable devices, and mobile applications have also optimized care, particularly in regions with limited infrastructure. However, challenges remain concerning the validation of algorithms across diverse populations, the inclusion of sociodemographic variables, and ethical considerations. In conclusion, AI is a promising technology in obstetric care, with the potential to reduce maternal morbidity and mortality. Nevertheless, its implementation requires ethical guidelines, adequate professional training, and inclusive public policies that promote digital equity in maternal and child healthcare.

## 1 INTRODUCTION

Maternal mortality remains one of the main indicators of public health quality worldwide, reflecting the living conditions of women, access to health services, and the effectiveness of obstetric care policies. Despite global advances, it continues to be an avoidable cause of death that disproportionately affects low‐ and middle‐income countries. According to the WHO, maternal mortality is defined as the death of a woman during pregnancy or up to 42 days after its end, due to causes related to or aggravated by the pregnancy or its management, excluding accidental causes.^1^

The concept of near miss or maternal near death refers to women who have survived serious complications during pregnancy, childbirth or the puerperium, but who were at imminent risk of death. Analyzing these cases has proved to be an important strategy for understanding failures in health systems and proposing improvements in obstetric care, since the determinants of near miss are often the same as those of maternal deaths.^2^

In Brazil, maternal mortality continues to be a critical public health problem. Although the country has committed to reducing the maternal mortality ratio to fewer than 30 deaths per 100 000 live births by 2030, the most recent data from the Ministry of Health indicate rates far above this target. In 2021, the maternal mortality ratio reached 107.53 deaths per 100 000 live births, almost doubling the rate of 2019, largely due to the impact of the COVID‐19 pandemic.^1^, ^2^, ^3^, ^4^ Although there was a reduction to 57 deaths per 100 000 live births in 2022, regional and social disparities remain significant. The North and Northeast regions present the highest mortality ratios, while Black, Indigenous, and low‐educated women are disproportionately affected, underscoring the role of structural barriers in accessing timely and quality obstetric care.^3^, ^5^, ^6^

The main causes of maternal death in Brazil are mostly preventable and include hypertensive complications, hemorrhages, infections, and unsafe abortions. These events often result from failures in early risk identification, prenatal follow‐up, and clinical decision making. Studies show that inadequate prenatal care, delays in care during obstetric emergencies, and the fragility of surveillance systems are central to the persistence of these outcomes.^2^, ^7^, ^8^ Additionally, institutional racism, socioeconomic inequities, and underreporting of maternal deaths, especially those occurring outside hospitals or misclassified on death certificates, contribute to the invisibility and underestimation of the problem.^9^, ^10^

In this context, artificial intelligence (AI) emerges as a promising technological tool with potential to optimize clinical care, support decision making, and improve maternal and neonatal outcomes. International studies have demonstrated the ability of AI to identify complex patterns in large health datasets and anticipate adverse events such as pre‐eclampsia, postpartum hemorrhage, and preterm birth.^11^, ^12^ These technologies include machine learning (ML) algorithms, decision support systems, and wearable devices that assist healthcare professionals in real‐time.^13^, ^14^ Nonetheless, while the global context has seen rapid advances in the adoption of AI, especially in countries with robust digital infrastructure, the Brazilian scenario presents additional challenges related to technological inequality, limited integration of digital systems, and regional disparities in resource availability.^6^, ^10^

The urgency of adopting AI‐based tools in Brazil must be understood within the country's epidemiological and structural specificities. Rather than simply reproducing global models, it is necessary to develop inclusive and context‐sensitive applications that respond to the realities of the Unified Health System (SUS). This includes validating algorithms in diverse populations, ensuring representativeness of data, and promoting digital equity in maternal care.^5^, ^9^, ^11^

Although the use of AI in scientific production and health care raises important ethical questions, such as authorship, responsibility, and the risk of bias, these aspects will be addressed later in the discussion, in light of the literature reviewed. The academic debate has emphasized that AI tools, when misused, can generate content that appears methodologically sound but lacks scientific rigor, reinforcing the need for critical, ethical, and transparent use of these technologies.^11^, ^12^, ^13^ In educational and scientific contexts, there is a growing call for institutional guidelines that regulate the ethical application of AI tools and prevent their indiscriminate use.^12^

In this study, the distinction between the urgency of using AI in global and regional contexts, especially in Brazil, is addressed implicitly, but deserves to be more clearly demarcated. At a global level, AI has been increasingly incorporated into health systems as a strategy to improve care, reduce costs and increase diagnostic accuracy, especially in high‐income countries, where there is consolidated digital infrastructure, interoperability between systems and continuous investment in technological innovation. In Brazil, although maternal mortality continues to be a serious public health problem, with rates higher than the target recommended by the WHO, the adoption of AI‐based technologies is still limited and faces structural challenges, such as inequality in access to the internet, fragmentation of health data and the shortage of trained human resources. Thus, the urgency in Brazil lies not only in incorporating these technologies, but mainly in overcoming the barriers that limit their implementation in contexts of greater social and territorial vulnerability. Therefore, when considering AI as a tool to reduce preventable deaths, it is essential to situate its applicability and effectiveness based on the specificities of the Brazilian public health system. Finally, the lack of systematic syntheses focused specifically on the use of AI to reduce maternal mortality and near miss limits the advancement of scientific knowledge and the planning of evidence‐based policies.

## 2 METHODS

The present study is a narrative review of the literature, with a qualitative approach and exploratory‐interpretative. The choice of narrative review is justified by the complexity of the subject under investigation and the need to integrate heterogeneous studies in terms of methodological design, populations, implementation contexts and types of technology, respecting the plurality of approaches inherent in the subject.

This review followed the five structuring axes proposed by Rother^15^ and Greenhalgh et al.^16^ for narrative reviews: (1) Formulation of the research question; (2) delimitation of eligibility criteria; (3) definition of information sources and search strategies; (4) selection, extraction and critical analysis of data; and (5) interpretative synthesis with construction of emerging thematic categories.^15^, ^16^ The review was developed in accordance with the methodological recommendations of the Johns Hopkins University School of Public Health for narrative reviews and based on the principles of evidence‐based practice in health.

The guiding question of the review was: What is the scientific evidence on the application of technologies based on AI in gynecologic and obstetric health? The target population was composed of women in the pregnancy‐puerperal cycle; the interest was in AI tools applied in gynecologic and obstetric care and the context was the health care system at a global level.

The guiding question was: What is the scientific evidence on the application of technologies based on AI in gynecologic and obstetric health? Regarding the eligibility criteria, articles were included if they: (a) Directly address the use of AI technologies (such as ML, deep learning, neural networks, expert systems or natural language processing) in the context of gynecologic, obstetric, prenatal, childbirth, puerperal or maternal‐fetal monitoring care; (b) present as primary or secondary outcomes maternal health indicators, such as mortality, severe morbidity (near miss), early identification of risks, support for clinical decision making or improvement in the quality of care; (c) adopt empirical methodological designs, including clinical trials, observational studies (cohorts, case‐controls, cross‐sectional), methodological studies, as well as systematic or integrative reviews and evidence‐based guidelines; (d) were published between January 2019 and June 2025, a period that includes the intensification of the use of AI in health; and (e) were available in Portuguese, English or Spanish, with access to the full text through recognized scientific databases. The following were excluded: (a) Studies that addressed populations outside the pregnancy‐puerperal cycle, such as isolated newborns, health professionals or patients with other conditions that do not directly affect maternal health; (b) works that did not present clinical or operational outcomes directly related to women's health or obstetric care; (c) articles without peer review, such as technical reports, unreviewed preprints, dissertations, theses, editorials, letters or comments; and (d) studies focusing exclusively on AI applications in other medical areas or with an exclusively technical approach, without clinical implications for reproductive and maternal health.

The search was conducted between May and June 2025 in four electronic databases selected for their comprehensiveness and relevance in the areas of health and applied computer science: Literatura Latino‐Americana e do Caribe em Ciências da Saúde (LILACS), Medical Literature Analysis and Retrieval System Online (MEDLINE)/National Library of Medicine's (PUBMED), Scopus e Web of Science. The search strategy combined controlled descriptors and free keywords, articulated by Boolean operators AND and OR. The main terms used were: (“artificial intelligence” OR “machine learning” OR “deep learning” OR “natural language processing” OR “neural networks” OR “clinical decision support systems” OR “predictive models”) AND (“obstetric care” OR “gynecologic care” OR “prenatal care” OR “antenatal care” OR “maternal health” OR “pregnancy complications” OR “labor and delivery” OR “puerperium” OR “postpartum care” OR “maternal mortality” OR “maternal morbidity”). The search was complemented by exploring the reference lists of the included articles (reverse search) and by manual analysis of the main journals in the area.

The screening of articles was conducted in two stages: reading of titles and abstracts, carried out by two reviewers independently, in order to identify potentially eligible articles; full reading of the selected texts, with application of the inclusion and exclusion criteria. The extracted data were analyzed using narrative thematic synthesis, as proposed by Popay et al.^17^ The analysis sought to articulate the findings with theoretical frameworks of public health, bioethics and technological innovation in health. Regarding the reliability of the study selection process, the screening was performed independently by two reviewers. To ensure consistency of decisions, the inter‐rater agreement index was calculated using Cohen's kappa coefficient, which resulted in a value of 0.84, indicating high agreement (values above 0.80 are considered excellent). This result reinforces the reliability of the inclusion and exclusion process of the analyzed studies.

The software Interface de R pour les Analyses Multidimensionnelles de Textes et de Questionnaires (IRaMuTeQ) was used to organize and present the results of the narrative review. This is a free program, developed based on the statistical language R and the Python platform, which allows statistical analyses of textual corpora and data from questionnaires. Among the functionalities offered by IRaMuTeQ, the following stand out: similarity analysis, word cloud, descending hierarchical classification, as well as correspondence factor analyses and content analyses with statistical support. The use of IRaMuTeQ in this narrative review allowed an in‐depth reading of the texts, identifying thematic groupings and lexical recurrences that contributed to the interpretation of the data in a more objective and structured way.^18^

Regarding the textual analysis performed with the IRaMuTeQ software, although the tool has methodological recognition in the analysis of qualitative data and a solid statistical basis (frequency, chi‐square, lexical grouping and factor analysis), a formal test of external validity or reproducibility of the results generated was not performed. However, the emerging categories and lexical classes obtained in the analyses were manually reviewed by two researchers, with the aim of verifying the semantic coherence of the groupings and ensuring the reliability of the interpretation.

## 3 RESULTS

**FIGURE 1:**
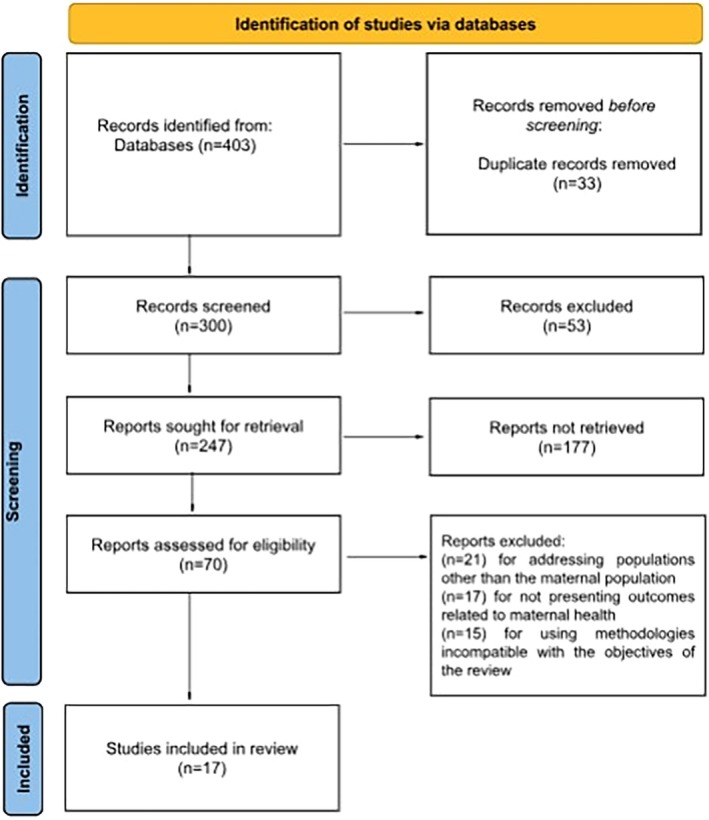
Flow chart of the study selection process according to the Preferred Reporting Items for Systematic Reviews and Meta‐Analyses (PRISMA). Source: Author. 2025.

The initial search identified (*n* = 392) records in the electronic databases, to which were added (*n* = 11) additional articles located through manual search and analysis of cross‐references, totaling (*n* = 403) records. After removing duplicates (*n* = 370) unique articles remained for screening. Reading the titles and abstracts resulted in the exclusion of (*n* = 300) studies because they did not meet the previously established eligibility criteria. The remaining (*n* = 70) articles were evaluated in full, and (*n* = 53) were excluded: (*n* = 21) for addressing populations other than the maternal population, (*n* = 17) for not presenting outcomes related to maternal health, and (*n* = 15) for using methodologies incompatible with the objectives of the review, as shown in Figure 1. At the end of the process, (*n* = 17) studies were included in the narrative synthesis, as illustrated in Figure 1, and analyzed with the support of the IRaMuTeQ software.

The use of IRaMuTeQ software contributed to the interpretation of the theme by offering an automated, objective and statistically based textual analysis, which complements and, in many aspects, deepens the manual coding. IRaMuTeQ made it possible to process large volumes of text more quickly, identifying linguistic patterns, word frequencies, co‐occurrences and thematic groupings through techniques such as descending hierarchical classification (DHC) and similarity analysis. In addition, IRaMuTeQ allows the data to be visualized graphically, which facilitates the understanding of the relationships between terms and emerging categories. Thus, the integration between manual coding and analysis via IRaMuTeQ enriches the interpretation of the material, offering a methodological triangulation that increases the validity of the findings and strengthens the scientific discussion.

The articles included in the review were published between 2020 and 2025, predominantly in English, with some publications in Portuguese, and originated from different international contexts, including high‐ and low‐income countries. The main objective of the studies was to investigate the applications of AI in maternal health, nursing, and obstetrics, with a focus on gestational assessment, early prediction of complications, clinical decision support, and reduction of maternal mortality. The methodologies employed included systematic reviews, meta‐analyses, original quantitative studies on algorithm development and validation, and narrative reviews. The level of evidence ranged from moderate to high, mainly in systematic reviews and technology validation studies. Overall, the synthesis shows that AI has proven to be a promising tool for improving diagnosis, monitoring, and clinical decision making, especially in resource‐limited settings, although there is a need to expand clinical validation and the integration of these technologies into daily practice, as shown in Table 1.

**TABLE 1:** Summary of studies included in the review on AI in women's health.

Year	Author/title	Type of study/level of evidence	Objectives	Main results
2025	Tzimourta et al.^30^ Maternal Health Risk Detection: Advancing Midwifery with Artificial Intelligence	Level 2 Diagnostic/prognostic accuracy study using ML	Relating high, low, and medium‐risk maternal health using machine learning algorithms based on physiological data	The best performance was found for Random Forest, also achieving the highest values of accuracy (88.03%) and precision (88.10%), showing its robustness in handling maternal health risk classification. The medium risk category was the most challenging in all models, characterized by lower recall and precision scores, thus highlighting class imbalance as one of the bottlenecks in performance
2025	Owoche et al.^32^ The role of AI in reducing maternal mortality: Current impacts and future potentials: Protocol for an analytical cross‐sectional study	Level 5—study protocol for a mixed‐methods crosssectional analysis	To evaluate the implementation, user experiences and impact of OPOCUS and PROMPTS on maternal and newborn health outcomes in Kenya	The study generated empirical evidence on the feasibility, effectiveness, and barriers to integrating AI into maternal health services. The results propose policy recommendations, expand the design of AI‐assisted maternal health care, and support the scaling of AI‐based interventions to improve maternal and newborn health outcomes in Kenya and other resource‐poor settings
2023	Rosa^22^ Inteligência artificial na enfermagem: aplicações e benefícios para a prática profissional	Level 3/4 Expert opinion and theoretical reflection based on literature review	Carry out a bibliographic review on the applicability of AI in obstetric nursing, highlighting its main functionalities and benefits	The results indicate that AI can assist nurses in a variety of activities, such as patient support and triage, monitoring, medication management, planning, care coordination, documentation assistance, education, data analysis, forecasting, and assistance with certain procedures
2024	Kim^23^ The effects of artificial intelligence chatbots on women's health: a systematic review and meta‐analysis	Systematic review and meta‐analysis Level 1—high (RS + meta‐analysis)	To investigate the effects of AI chatbot interventions on health outcomes in women	Chatbots were effective in addressing anxiety, depression, grief, healthy relationships, cancer self‐care behavior, preconception intentions, risk perception in eating disorders, and gender attitudes. Chatbot users showed benefits in terms of internalization, acceptability, feasibility, and interaction
2024	Lin et al.^25^ Artificial intelligence‐augmented clinical decision support systems for pregnancy care: systematic review	Systematic review and meta‐analysis Level 1—high (RS + meta‐analysis)	Identify and synthesize AI‐augmented CDSS in pregnancy care, CDSS functionality, AI methodologies, and clinical implementation	Clinical application topics covered AI‐enhanced CDSS from prenatal, early pregnancy, obstetric care, and postpartum care. CDSS function topics include diagnostic support, clinical prediction, therapeutic recommendation, and knowledge base
2024	Quixabeira et al.^33^ Métodos de inteligência artificial na predição e diagnóstico precoces de complicações na gravidez	Integrative review Level 3—moderate (integrative review with clear criteria)	To analyze and synthesize the scientific evidence available in the literature on AI methods for the early prediction and diagnosis of pregnancy complications	The study highlights the significant impact of AI models in improving the prediction and diagnosis of pregnancy complications. The results highlight the need for continued integration of these technologies into obstetric clinical practice
2024	Oliveira et al.^34^ Abordagens e eficácia da Inteligência Artificial na predição de hemorragia pós‐parto	Integrative review Level 3—moderate (integrative review with clear criteria)	Identify approaches and effectiveness of AI in predicting PPH	The predominant research designs were cohort studies. The AI models demonstrated high accuracy and sensitivity, excelling in integrating multiple predictive factors for PPH, such as maternal pre‐gestational weight, gestational age, gestational hypertension, and uterine contraction characteristics
2023	Mennickent et al.^31^ Machine learning applied in maternal and fetal health: a narrative review focused on pregnancy diseases and complications	Integrative review Level 3—moderate (integrative review with clear criteria)	Describe the state of the art on the use of AI in the context of diseases and gestational complications	The use of AI in gestational diseases and complications is quite recent and has increased in recent years. The applications are varied and aim not only at diagnosis, but also at management, treatment and pathophysiological understanding of perinatal changes
2022	Silva Rocha et al.^19^ On usage of artificial intelligence for predicting mortality during and post‐pregnancy: a systematic review of literature	Systematic review and meta‐analysis Level 1—high (RS + meta‐analysis)	To present a systematic review of the literature focused on computational models for mortality prediction, covering maternal and perinatal deaths	It was found that most of the work focuses on predicting deaths, using machine learning models (more specifically Random Forest). Having predictive models to prevent mortality during and after pregnancy not only improves the mother's quality of life, but can also be a powerful and low‐cost tool to reduce mortality rates
2022	Gomes et al.^20^ A mobile‐optimized artificial intelligence system for gestational age and fetal malpresentation assessment	Level 2—prospective diagnostic accuracy study	Investigating the use of artificial intelligence for obstetric ultrasound in low‐resource settings	The approach uses artificial intelligence to automatically interpret ultrasound video. The system consists of an ultrasound device and can operate without internet connectivity, making it suitable for deployment in low‐resource environments
2022	Gomes et al.^21^ AI system for fetal ultrasound in low‐resource settings	Level 2—prospective diagnostic accuracy study	Develop and validate an AI system that uses ultrasound videos	Using a simplified scanning protocol with real‐time AI feedback on scan quality, demonstrated generalizability of model performance to novice ultrasound operators with minimal training, using low‐cost ultrasound devices with on‐device AI integration
2022	Von Gerich et al.^26^ Artificial intelligence‐based technologies in nursing: a scoping literature review of the evidence	Narrative review Level 6—low (reflective narrative)	To synthesize the cutting‐edge research currently available in artificial intelligence‐based technologies applied to nursing practice in women's health	Most studies explored the technology development (*n* = 55, 59.1%) and training (testing) (*n* = 28, 30.1%) phases, followed by the implementation (*n* = 9, 9.7%) and operational (*n* = 1, 1.1%) phases. The vast majority (73.1%) of studies provided evidence with a descriptive design (level VI), while only a small portion (4.3%) were randomized controlled trials (level II)
2022	Khan et al.^29^ On AI Approaches for Promoting Maternal and Neonatal Health in Low Resource Settings: A Review	Narrative review Lev el 6—low	Present an analysis of digital solutions for maternal and newborn health in low‐resource settings and discuss open issues as well as future research directions	With the advent of AI, digital technologies have emerged as practical tools for assistance in different health sectors, but they are still in their early stages when applied to maternal and newborn health
2021	Ramakrishnan et al.^27^ Perinatal health predictors using artificial intelligence: A review	Narrative review Level 6—low	Provide an overview of maternal and perinatal health indicators, summarize the evidence on the current state of evidence for the application of AI	The potential of AI‐based algorithms and methods for developing new prediction models, improved diagnosis, early identification, and monitoring of women during pregnancy, childbirth, and postpartum to advance research, clinical practice, and policy, and ensure optimal perinatal health, was summarized
2021	Nistal‐Nuño^35^ Artificial intelligence predicting mortality in an intensive care unit and comparison with a logistic regression system	Level 2 Diagnostic/prognostic accuracy study using machine learning	To explore an AI approach based on gradient‐boosted decision trees for predicting all‐cause mortality in intensive care units, comparing its performance with a recent logistic regression system in the literature and a logistic regression model	The gradient‐boosted decision tree model outperformed the logistic regression model in predicting 12 h mortality in intensive care units. The excellent performance of gradient‐boosted trees was achieved despite the cohort being an unbalanced dataset, highlighting the usability and flexibility of AI models
2020	Jeong^24^ Artificial intelligence, machine learning, and deep learning in women's health nursing	Level 6—low (reflective narrative)	Analyze the concepts of AI, need for AI education in nursing schools; and areas of nursing care where AI is useful	An example of an area of nursing care where AI is useful is prenatal nursing interventions based on nursing records of pregnant women and AI‐based prediction of birth risk according to the age of pregnant women
2020	Iftikhar et al.^28^ Artificial Intelligence: A New Paradigm in Obstetrics and Gynecology Research and Clinical Practice	Narrative review Level 6—low (reflective narrative)	Address current uses of AI as a tool to interpret fetal heart rate and cardiotocography to aid in the detection of preterm labor, pregnancy complications, and review discrepancies in their interpretation to reduce maternal and infant morbidity and mortality	The benefits of using AI's vast data storage capacity can help determine risk factors for preterm labor using multi‐omics and extensive genomic data. In the field of gynecologic surgery, the use of augmented reality helps surgeons detect vital structures, thereby decreasing complications, reducing operative time, and helping surgeons‐in‐training practice in a realistic environment

Abbreviations: AI, artificial intelligence; CDSS, clinical decision support systems; ML, machine learning; PPH, postpartum hemorrhage; RS, systematic review.

**FIGURE 2:**
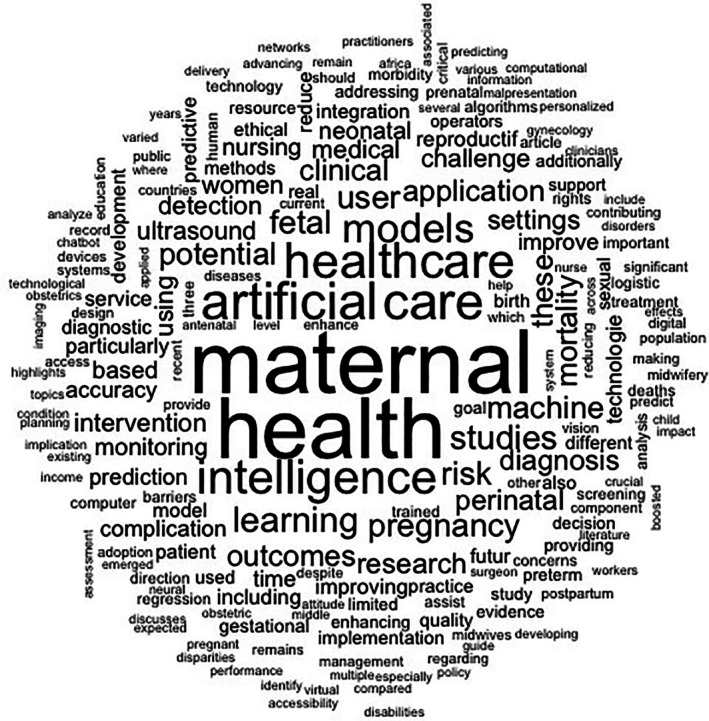
Key themes in the use of artificial intelligence in gynecologic and obstetric healthcare. Source: Author. 2025.

The word cloud reveals that the use of AI in gynecologic and obstetric healthcare is primarily centered on maternal and infant care, with emphasis on terms such as “maternal,” “health,” “care,” “pregnancy,” “perinatal,” and “neonatal.” The prominence of words like “artificial,” “intelligence,” “learning,” and “models” underscores the focus on applying advanced technologies, particularly ML models to optimize healthcare delivery. Key applications of AI include pregnancy monitoring, diagnosis, and risk prediction, as indicated by terms such as “monitoring,” “diagnosis,” “prediction,” “detection,” and “screening.” Technologies like ultrasound and fetal outcome analysis (“ultrasound,” “fetal,” “outcomes,” “complication”) also emerge as practical areas of implementation. The context is predominantly clinical and hospital‐based, as shown by words like “clinical,” “healthcare,” and “medical,” involving both healthcare providers, including physicians and midwives (“providers,” “midwives”) and patients (“user,” “patients”). At the same time, the word cloud highlights critical challenges in implementing AI, with attention to issues such as accuracy, bias, accessibility, disparities, and policy. The inclusion of terms like “resource,” “low‐income,” and “limited” suggests a concern with deploying these technologies in low‐resource settings, pointing to the need for equity in access. Terms such as “postpartum,” “gestational,” “preterm,” and “mortality” further reinforce the potential role of AI in preventing complications and reducing maternal and neonatal morbidity and mortality. In summary, the word cloud indicates that AI holds promise for transforming gynecologic and obstetric care but also calls for thoughtful consideration of its technical, ethical, and social challenges, as shown in Figure 2.

The semantic similarity graph illustrates the key conceptual connections present in a narrative review article on the use of AI in gynecologic and obstetric healthcare. At the center of the graph, terms such as “artificial intelligence,” “healthcare,” “models,” and “clinical” highlight that the core focus of the review is the clinical application of AI models in healthcare, particularly in pregnancy and childbirth contexts. From this central cluster, several interconnected thematic groups emerge.

One of the main clusters relates to the practical applications of AI, featuring terms like “diagnosis,” “detection,” “pregnancy,” “ultrasound,” “fetal,” and “gestational,” which reflect the use of AI technologies for early diagnosis, fetal anomaly detection, and pregnancy monitoring, especially through tools such as ultrasound. Another significant grouping focuses on clinical outcomes, with terms such as “maternal,” “infant,” “mortality,” “intervention,” “outcomes,” “birth,” and “complication.” This suggests that AI is being used to improve care quality, prevent obstetric complications, and reduce maternal and neonatal morbidity and mortality. A separate cluster of technical terms such as “regression,” “logistic,” “gradient,” “boosting,” “personalized,” “predictive,” and “systems” demonstrates the article's engagement with the statistical methods and algorithms used in AI model development. In parallel, a group of terms related to implementation challenges includes “implementation,” “accuracy,” “ethics,” “access,” “limited,” “low‐income,” “policy,” and “bias.” These words point to structural and ethical concerns discussed in the article, such as algorithmic bias, regulatory gaps, model accuracy, and inequalities in access to AI in low‐resource settings.

**FIGURE 3:**
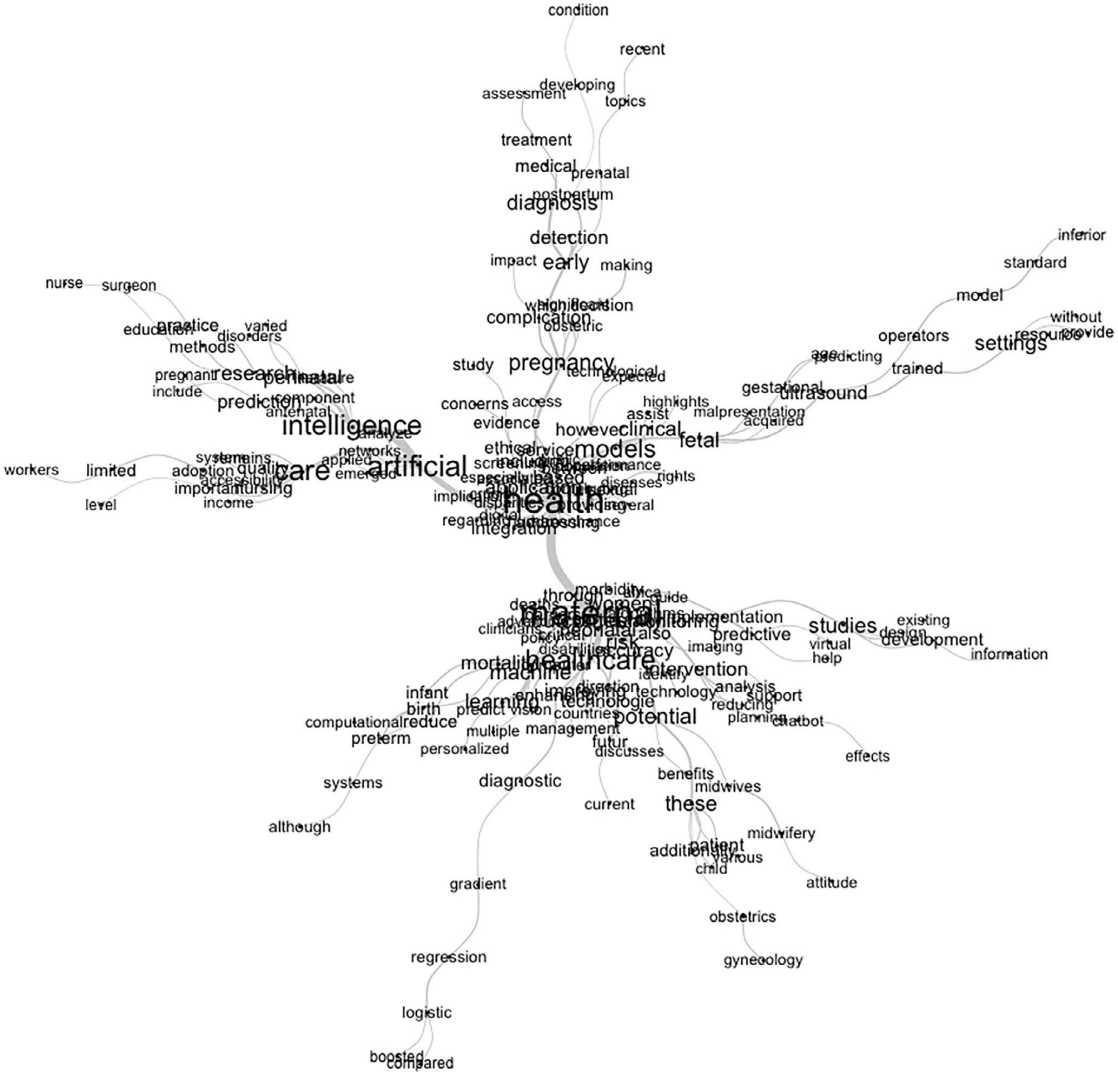
Conceptual network of artificial intelligence applications in maternal and reproductive health. Source: Author. 2025.

Another important cluster relates to the healthcare workforce, with terms like “midwives,” “providers,” “education,” “training,” “nurse,” “surgeon,” and “workers.” This reflects the need for training, professional development, and the inclusion of healthcare workers in the safe and effective adoption of AI. Finally, the graph reveals a group focused on critical and methodological reflection, with terms such as “research,” “evidence,” “study,” “practice,” “methods,” and “analysis,” indicating that the article reviews scientific evidence, examines methodologies, and discusses clinical practices in the light of technological innovation, as shown in Figure 3.

The dendrogram reveals two prominent thematic classes emerging from the textual analysis of a narrative review on the use of AI in gynecologic and obstetric healthcare. These classes represent distinct lexical and conceptual fields within the corpus.

Class 2, which accounts for 29.3% of the analyzed content, centers around the theme of healthcare enhancement and the implementation challenges associated with AI. Words like “enhancing,” “healthcare,” “rights,” “sexual,” “practitioners,” “costs,” “implementation,” and “technology” suggest that this class is concerned with how AI can be harnessed to improve the quality, equity, and accessibility of reproductive and sexual healthcare services. It reflects an ethical and practical perspective, emphasizing the potential of AI to reduce disparities, support clinical professionals, and respect patients' rights. The inclusion of terms such as “implication,” “reduce,” and “target” indicates a focus on translating technological advancements into concrete benefits for healthcare delivery, especially in under‐resourced or vulnerable populations.

Class 3, comprising 28% of the content, is more technical in nature, focusing on the diversity and limitations of AI models in this field. It includes words such as “models,” “user,” “learning,” “machine,” “algorithms,” “multiple,” “varied,” and “different,” pointing to a critical discussion on the nature of ML systems. The presence of connectives like “but,” “not,” and “from” suggests a reflective tone, likely addressing issues such as algorithmic diversity, model generalizability, and user adaptability. This class appears to emphasize the complexity and heterogeneity of AI tools, highlighting that not all models are equally effective across different clinical contexts or patient populations.

**FIGURE 4:**
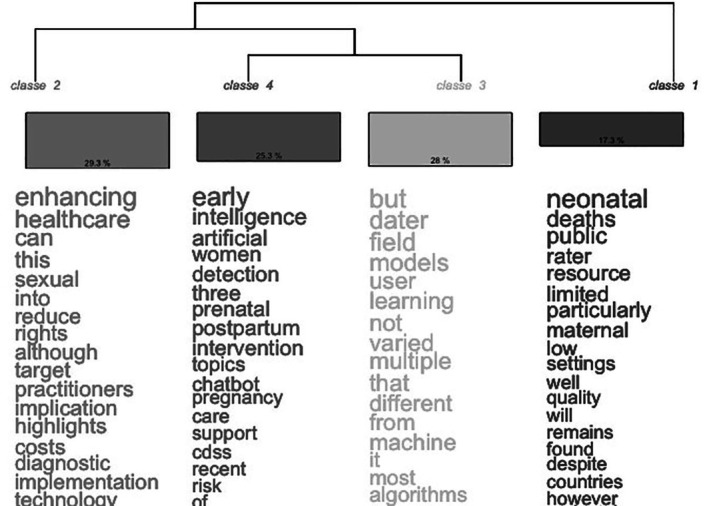
Thematic lexical classification of artificial intelligence discourse in reproductive healthcare literature. Source: Author. 2025.

Taken together, these two classes illustrate a dual focus in the narrative review: one grounded in ethical, clinical, and implementation‐related considerations, and the other in technical and methodological analysis. This balance reflects a comprehensive approach to understanding AI's role in gynecologic and obstetric healthcare, recognizing both its transformative potential and the limitations that must be addressed to ensure safe, equitable, and effective use, as shown in Figure 4.

The data presented are represented by weights or coefficients associated with four different classes for a series of words related to the context of maternal health, AI and ML. Each word has a value for each of the four classes, which indicates the importance or association of that word with the respective class, as shown in Table 1.

In class 1, the words with the highest positive weight include terms such as maternal (13261), neonatal (31623), settings (8539), low (11077), rater (14904), resource (14904), limited (14904), particularly (14682), deaths (20152), and public (20152). This suggests that this class is strongly associated with clinical and epidemiological aspects of maternal and neonatal care, including limited resources, mortality, and care settings.

In class 2, words related to technology and ML stand out, such as healthcare (13269), this (10145), using (7528), improving (6.6), integration (1921), diagnostic (6634), sexual (9174), enhancing (15711), rights (7528), education (4.63), reproductive (6.6), and years (7528). This indicates that class 2 represents themes related to the application of AI and ML in the health area, highlighting technological integration, diagnostics, education, and reproductive rights.

Class 3 is marked by positive values in terms such as dater (10096), models (8774), learning (8, 12), from (7185), different (7223), not (8036), varied (8036), topics (9211), chatbot (9211) and ML (19853). This suggests that class 3 is related to the development and use of ML models, algorithms, user interaction and automated systems, such as chatbots.

Finally, class 4 has positive weights in words such as artificial (14045), intelligence (15602), early (26394), care (5577), pregnancy (5815), intervention (11679), detection (12454), women (12454), postpartum (12454), three (12454), and prenatal (12454). This class appears to focus on the early application of AI for clinical intervention, monitoring, and detection in maternal health, especially during prenatal and postpartum, highlighting direct care for women, as shown in Table 2.

**TABLE 2:** Distribution of relevant terms by thematic classes in maternal health and AI.

Class	Central theme	Relevant words	Interpretation
1	Maternal and neonatal mortality	Neonatal, deaths, public, maternal	Focus on structural problems and inequalities in maternal health
2	Technological implementation	Healthcare, enhancing, improving, rights	Integration of technologies into health policies and services
3	AI techniques	ML, models, learning, algorithms	Technical discourse on AI model development and variation
4	Clinical applications	Artificial, intelligence, care, pregnancy	Practical use of AI in diagnostics, interventions and clinical monitoring

Abbreviations: AI, artificial intelligence; ML, machine learning.

## 4 DISCUSSION

The integration of AI into scientific and educational practices has sparked relevant debates about ethics, authorship, responsibility and the quality of the knowledge produced. In the academic context, the use of these tools still lacks clear regulations. Artificial intelligence can be an ally in the writing of scientific texts, but it should not be recognized as an author, since it lacks intentionality, creativity and ethical responsibility. The central concern lies in the indiscriminate use of AI, which can compromise the integrity of scientific production by creating content that appears legitimate but lacks an empirical basis and methodological rigor.^11^

In line with this, the preprint discusses the potential of AI in academic production and in democratizing access to information. The authors point out that although the tool has advantages in terms of agility and textual organization, its use raises challenges in terms of originality, quality of sources and the possibility of plagiarism. The study recommends the formulation of institutional guidelines to guide its ethical and responsible use, suggesting that it be used as a support and not as a substitute for critical and reflective scientific work.^12^

In the field of health, Gaceta Médica de México offers a perspective on the use of AI to improve diagnoses and the management of health services. The article stresses that although tools based on AI can help systematize clinical information and decision making, their use must be cautious, considering the risks of interpretative errors and the need for validation by qualified professionals. The text warns against the false sense of precision that AI can convey, especially when its responses are not subjected to technical verification.^13^

An integrative analysis carried out by the authors identified 392 studies that show the use of AI on various care fronts, such as predicting and preventing adverse events, early identification of clinical changes and data‐based decision making. Among the most frequently addressed adverse events are hospital‐acquired infections, medication errors, thromboembolism, surgical complications, pressure ulcers, falls, clinical deterioration and inaccurate diagnoses. The results show that the most widely used tools are ML algorithms, wearable sensors, computer vision systems and clinical decision support platforms. These technologies enable continuous, real‐time monitoring of patients, the integration and analysis of large volumes of data and the issuing of automated alerts for imminent risks, which enhances the preventive action of healthcare teams. Another relevant finding was that AI systems are more effective in environments with a high volume of data and where clinical complexity makes it difficult to identify risk patterns manually. Artificial intelligence proved effective, for example, in anticipating episodes of sepsis, identifying patients at high risk of falling and suggesting personalized adjustments to therapeutic plans, reducing human error and improving clinical outcomes.^14^

Artificial intelligence has established itself as a promising tool for improving maternal health, especially in low‐ and middle‐income contexts where maternal mortality and near misses remain high. The ability of AI to analyze large volumes of data and identify complex patterns allows for the early detection of risks, facilitating timely and effective interventions. Studies show that ML models, such as Random Forest, can predict adverse pregnancy outcomes using variables such as birth weight, gestational age and maternal age.^19^

In addition, AI has been successfully applied in resource‐limited environments. For example, AI systems integrated with portable ultrasound devices have helped health professionals with limited training to estimate gestational age and identify abnormal fetal presentations, improving prenatal care. Artificial insemination‐based mobile applications have also been developed to support midwives during home visits, offering real‐time analysis and feedback based on collected data, as demonstrated in initiatives in Guatemala.^20^, ^21^

Artificial intelligence has been applied in various areas of nursing, providing significant advances in women's health. Machine learning algorithms help with patient triage, vital signs monitoring and medication management, optimizing care planning and coordination. Tools such as DeepMind Health and IBM Watson Health exemplify these applications, improving the quality of care and patient satisfaction.^22^

For example, AI‐based chatbots have been shown to be effective in reducing symptoms of anxiety and depression, as well as promoting self‐care behaviors in women. A systematic review and meta‐analysis identified that these digital interventions are feasible and well accepted, representing a form of digital therapy to support women's health. In addition, AI‐enhanced clinical decision support systems have been developed for antenatal care. These systems assist healthcare professionals in the early identification of risks during pregnancy, improving clinical decision making and potentially reducing maternal and neonatal complications.^23^

A study highlights several applications of AI, such as predicting risks during pregnancy based on nursing records and maternal age. These tools help with clinical decision making, improving the quality of care provided to women. The effective implementation of AI in nursing requires professionals to be adequately trained. There is a need to include content on AI, MLand big data analysis in nursing curricula. This training is essential so that nurses can understand and apply these technologies ethically and effectively in their daily practice.^24^

A systematic review shows that AI‐enhanced clinical decision support systems (CDSS) have been increasingly applied in prenatal care, with emphasis on four main areas of action: diagnostic support, clinical prediction, therapeutic recommendations and the development of knowledge bases. The review identified 30 empirical studies that used different AI methodologies, such as ML, neural networks and rule‐based systems, to improve decision making during pregnancy. The CDSS analyzed were used in a variety of clinical contexts, including the early diagnosis of fetal anomalies, support for ultrasound examinations, gestational risk assessment and the formulation of personalized therapeutic plans. They have also been used to create clinical ontologies capable of organizing and interpreting large volumes of obstetric data, with the aim of supporting more accurate and effective medical decisions.^25^

The application of AI‐based technologies in nursing is growing and diversifying, with a greater presence in the areas of clinical decision support, patient monitoring, care management and education. Most of the studies reviewed described AI tools used to support nurses in decision making, especially in the early identification of clinical risks, such as deterioration of the patient's condition, and in defining more precise therapeutic conducts.^26^

Second, recent reviews, such as the one by Lin et al., show that although there are promising applications of AI in prenatal care, there are important gaps related to its actual effectiveness, integration into clinical practice and consideration of social and ethical factors.^25^ Similarly, studies such as that by von Gerich et al. reveal that, in nursing, most research is still focused on the systems development phase, with little evidence on clinical impact, implementation and the active participation of nursing professionals in the innovation process.^26^

Another relevant study reinforces the need to consider the ethical impacts of AI on maternal health, with an emphasis on confidentiality, patient autonomy and the risk of algorithmic discrimination. The research highlights that although digital tools can optimize obstetric surveillance, their implementation requires clear guidelines, international regulation and ethical education for health professionals.^27^, ^28^

Artificial insemination technologies can transform maternal‐fetal health care at all stages of pregnancy, highlighting that techniques such as ML, neural networks, computer vision and natural language processing have the potential to improve diagnosis, monitoring and clinical decision making throughout the pregnancy cycle. Artificial insemination can, for example, identify hidden patterns in large volumes of medical data and predict complications such as pre‐eclampsia, gestational diabetes and intrauterine growth restriction, more accurately and earlier than traditional methods. The use of computer vision applied to imaging examinations, such as ultrasounds, to detect fetal anomalies early and issue automatic alerts. In addition, AI‐based systems can monitor maternal and fetal vital parameters in real time, such as blood pressure and heart rate, increasing the safety of prenatal care and childbirth. Another highlight is the application of natural language processing to extract relevant information from electronic clinical notes, which increases the ability to identify risks even in unstructured records.^29^, ^30^

Additionally, the study by Mennickent et al. emphasizes the role of sociodemographic data in building more inclusive algorithms, pointing out that most of the models developed reflect populations in high‐income countries, which compromises their generalizability to other realities. Thus, the article proposes investing in local and collaborative databases to strengthen digital equity in maternal health.^31^

The incorporation of AI in maternal health has shown significant advances in various clinical contexts, as demonstrated by Owoche et al. who highlight the role of AI in reducing maternal and neonatal mortality through predictive systems, clinical assistants and remote monitoring. The study emphasizes that technologies such as ML and language models can improve clinical decision making and resource allocation in low‐income settings, although it also acknowledges important barriers such as data quality and interoperability between digital systems.^32^

An integrative review aimed at gathering scientific evidence on the application of AI tools in the early recognition of conditions that threaten maternal‐fetal health, the research analyzed 13 articles published between 2019 and 2023, mostly cohort studies developed in China, which used ML algorithms such as Random Forest, neural networks and support vector machines (SVM) to predict complications such as pre‐eclampsia, gestational diabetes, premature birth and abnormal placental insertion. The data used in these studies came from electronic medical records and routine laboratory tests, which reinforces the viability of the clinical use of these models. The results showed high accuracy in the early detection of these conditions, which could allow timely interventions and reduce maternal and neonatal morbidity and mortality. The authors point out, however, that the implementation of these technologies in obstetric practice depends on the validation of these algorithms in different population contexts and the adequacy of health structures, especially in low and medium complexity environments.^33^

Oliveira et al. presents an integrative review with the aim of identifying and analyzing the main AI approaches applied in the early prediction of postpartum hemorrhage (PPH), one of the leading causes of maternal mortality in the world. The research included six studies published between 2018 and 2024. Most of the studies were carried out in the USA and adopted ML algorithms such as Random Forest, artificial neural networks and support vector machines (SVM), fed with clinical data such as gestational age, parity, presence of gestational hypertension, pre‐gestational maternal weight and uterine contractility parameters. The models showed high sensitivity and specificity in identifying pregnant women at increased risk of PPH, suggesting that AI could be a promising tool for anticipating serious complications and improving obstetric outcomes. The authors point out that, although the findings are positive, more research is still needed to test the effectiveness of these algorithms in different clinical and population contexts, especially in low‐ and middle‐income countries, where the burden of maternal mortality is higher.^34^

A descriptive study evaluated the performance of AI models, in particular ML algorithms based on gradient‐boosted decision trees (GBDT), compared to traditional logistic regression models for predicting the mortality of patients admitted to intensive care units (ICUs). Using data from the MIMIC‐III database, which contains detailed clinical and physiological information on critically ill patients, the models were trained with data collected in the first 5 h of hospitalization, including vital signs and laboratory variables, and the outcome analyzed was mortality in the subsequent 12 h. The results showed that the GBDT model outperformed logistic regression in several metrics: area under the ROC curve (0.89 vs. 0.806), accuracy (81.4% vs. 78.2%) and diagnostic odds ratio (17.823 vs. 9.254). In addition, indicators such as the F‐score, the Matthews correlation coefficient and Cohen's kappa index also favored the AI‐based model. The study concludes that AI, especially with algorithms such as GBDT, can be a powerful tool to support clinical decision making in intensive care settings by providing more accurate and sensitive predictions than conventional statistical methods, especially in unbalanced databases.^35^

Bolarinwa et al. address the use of AI as a strategy to increase access to reproductive health for women with disabilities, particularly in African contexts. The study points out that wearable devices and virtual assistants can reduce physical and communication barriers, but warns of the need to develop inclusive public policies that ensure the equitable implementation of these technologies.^36^ Digital technologies can favor historically marginalized populations by providing remote monitoring and clinical alerts for health professionals. Despite the advantages, the study warns of inequalities in access to technology, which can deepen health inequities, especially in regions without adequate infrastructure.^37^

The use of AI in the early identification of gestational risks, such as premature birth, pre‐eclampsia and gestational diabetes. Machine learning models applied to large databases can predict the risk of premature birth with significant accuracy, for example, using algorithms such as XGBoost and recurrent neural networks to estimate the date of birth with up to 2 weeks of margin. In addition, deep learning systems applied to ultrasound examinations, cardiotocography and magnetic resonance imaging (MRI) have improved the accuracy and efficiency in the diagnosis of fetal anomalies. In the prediction of pre‐eclampsia and gestational diabetes, interpretable models such as explainable boosting machine (EBM) and cost‐sensitive neural networks have also shown effectiveness, identifying clinical risk factors such as previous hypertension and history of obstetric complications. For example, a gradient boosting model predicted early pre‐eclampsia, highlighting variables such as hypertension.^38^

In short, the central discussion revolves around five key areas of sexual and reproductive health where AI shows great potential: prenatal, perinatal, and neonatal care; contraceptive and infertility services; prevention of unsafe abortions; diagnosis and treatment of sexually transmitted infections; and cervical cancer screening. By analyzing large datasets, AI can optimize diagnoses, personalize treatments, perform early screening, and support telemedicine, especially in resource‐poor regions.^39^

Artificial intelligence, for example, can improve sperm, egg and embryo selection in assisted reproduction treatments, optimize menstrual cycles in reproductive health management applications and early detection of obstetric pathologies through ultrasound image analysis. Platforms such as chatbots also offer informational and emotional support, while predictive analytics systems identify women at risk of postpartum depression, adapting interventions based on individual data. Despite the opportunities, the article highlights important challenges. Ethical issues related to privacy, algorithm biases, reproductive sovereignty and data integrity are relevant concerns. In addition, AI has the risk of dehumanizing care, a critical factor in contexts that value emotional closeness and may be unfeasible in places with little technological infrastructure or specialized training.^39^, ^40^

Artificial intelligence has proven effective in reducing clinical errors through predictive models and real‐time monitoring systems, which directly contributes to patient safety and the quality of care provided. When properly integrated into practice, these resources can significantly improve clinical outcomes, especially in perinatal and neonatal settings. However, the study also highlights several barriers to the implementation of AI in this field. Ethical concerns, especially regarding data privacy and the possibility of bias in algorithms, are highlighted as important obstacles.^40^

One of the main challenges identified in this review concerns the external validation and generalization of AI algorithms in different contexts. Most of the models analyzed were developed based on data from high‐income countries, which compromises their applicability in different realities, such as those found in peripheral regions of Brazil or in low‐ and middle‐income countries.^31^, ^34^ This imbalance can result in algorithmic biases, reduced diagnostic accuracy, and even ethical risks related to equity of care.^27^, ^28^

Studies such as that by Mennickent et al. indicate that algorithms built with homogeneous data tend to be less effective in underrepresented populations, which highlights the need for investments in collaborative and localized databases, with representation of diverse sociodemographic profiles.^31^ In addition, initiatives such as that by Owoche et al., which propose cross‐sectional studies with validation in low‐income settings, demonstrate possible ways to increase the robustness and reliability of these systems on a global scale.^32^

The literature also suggests that the lack of standardized protocols for validating AI tools contributes to the heterogeneity of results and the difficulty of comparing studies.^25^, ^26^ Protocols should include multicenter trials with different populations, external temporal validation, assessment of model stability in different health systems, and measurement of ethical risks associated with the indiscriminate use of AI.^29^, ^30^ The implementation of open benchmarking platforms that allow the sharing and continuous evaluation of models by different research institutions and health services may represent a promising strategy to ensure the scalability and reliability of AI algorithms in maternal health.^31^, ^38^

For AI to be implemented ethically, safely, and effectively in diverse clinical settings, especially in resource‐limited settings, it is necessary to adopt a set of practical strategies that involve public policies, digital governance, professional training, and community participation. First, it is essential to establish clear regulatory frameworks that define criteria for security, data privacy, clinical responsibility, and algorithmic governance. Health agencies and public institutions should create multidisciplinary AI ethics review committees, with the participation of health professionals, technology experts, civil society representatives, and users of the health system.^27^, ^28^, ^31^

Second, it is necessary to ensure the transparency and auditability of algorithms. This includes the use of explainable AI technologies that allow professionals to understand how decisions are generated and to be able to challenge them when necessary, which is especially relevant in contexts with limited infrastructure and heterogeneous digital training.^29^, ^30^, ^38^ Another key strategy is the continuous training of health professionals. Continuing education programs should include topics such as digital ethics, data literacy, critical use of AI, and practices focused on humanized care. This training should be adapted to local realities, promoting not only technical skills but also cultural sensitivity and empathy.^24^, ^26^, ^28^

Furthermore, building local and collaborative databases is essential for developing context‐aware algorithms. This allows models to be trained and validated with representative data, reducing the risk of biases that can exacerbate existing inequalities.^31^, ^36^ Such initiatives should be accompanied by community oversight mechanisms and transparent informed consent on the use of health data.^27^, ^28^ The incorporation of AI should be guided by principles of equity, prioritizing its use in areas of greater vulnerability and associating technological innovation with strategies to strengthen the health system as a whole and not as a substitute for structural investments in human resources, equipment and essential supplies.^5^, ^6^, ^36^, ^37^

Although AI has the potential to transform obstetric care, its safe integration into clinical decision making in countries with limited infrastructure still faces significant challenges. In low‐ and middle‐income settings, the safe application of AI depends on multiple factors: the availability of quality data, interoperability between information systems, digital connectivity, and the training of health professionals to operate these technologies critically and ethically. Studies have shown that tools such as clinical decision support systems, predictive algorithms, and wearable devices have already been successfully applied in low‐resource settings, such as in regions of Africa and Latin America, contributing to early diagnosis and timely interventions.^20^, ^21^, ^32^, ^36^ However, most of these implementations still occur in controlled environments or in the pilot phase, which limits the generalization of their results for routine use in public health services with poor infrastructure.^25^, ^26^, ^34^

Furthermore, not all AI applications discussed in this review have been tested for effectiveness in real‐world settings. Many studies focus on the development and initial validation of algorithms using secondary or simulated datasets, especially in high‐income countries. Only a small proportion of the research has evaluated the clinical impact of AI tools in real‐world care settings, considering factors such as acceptance by health professionals, adaptation to clinical routine, and effects on maternal outcomes.^25^, ^26^, ^31^, ^34^ This highlights a gap in the scientific literature regarding the practical implementation and longitudinal impact assessment of these technologies. Thus, we highlight the urgent need for multicenter studies, conducted in different socioeconomic realities, that consider cultural, structural, and ethical aspects in the use of AI in maternal health.^5^, ^11^, ^31^, ^36^

In this sense, it is recommended that undergraduate and postgraduate courses in nursing, medicine, obstetrics and public health include, in their curricula, specific components on the fundamentals of AI, data science in health, digital ethics, and interpretation of predictive algorithms. The approach should be transdisciplinary, combining technical knowledge with critical analysis and bioethics, with attention to the particularities of the different levels of health care and regional vulnerabilities.^24^, ^26^, ^28^ In addition, continuing education programs should offer modular and ongoing training for professionals in the basic health network, maternity wards and specialized services, using active methodologies, clinical simulations and accessible digital platforms. Strategies such as short courses in partnership with public universities and federal institutes, regional workshops and hybrid training with remote support can expand the reach of these actions, especially in regions with limited infrastructure.^5^, ^36^, ^37^ This includes the creation of protocols for the ethical use of AI, guaranteeing transparency, data protection and clinical safety, as well as financing pilot projects that integrate technology and training in priority territories.^27^, ^31^, ^36^

This study presents several limitations inherent to the narrative review methodology. First, the lack of a systematic approach may introduce selection bias, as the inclusion of studies could have been influenced by the researchers' subjectivity. Additionally, the methodological diversity and heterogeneity of the contexts analyzed hinder direct comparisons between findings, limiting the generalizability of the results. Another important limitation concerns the scope of the databases consulted, which may not have captured the entirety of the scientific literature on the topic, especially studies not indexed or published in languages other than Portuguese, English, and Spanish. Furthermore, the exclusive focus on AI applications in gynecologic and obstetric care may have excluded interdisciplinary approaches relevant to comprehensive women's healthcare.

Although the studies included in this review demonstrate promising applications of AI in maternal health, it is essential to recognize that much of this evidence still has important limitations from a methodological and practical applicability perspective. A significant portion of the research analyzed is based on models developed with small sample sizes, retrospective datasets or electronic health records from single institutions, which limits the statistical robustness and generalizability of the findings.^25^, ^26^, ^33^

Furthermore, there is great heterogeneity in the criteria used to measure the accuracy of the algorithms, lack of cross‐validation and lack of standardization in the definition of maternal outcomes, which makes it difficult to compare studies and extrapolate results to different health systems.^24^, ^29^ The geographic concentration of scientific production in high‐income countries also represents a relevant bias, since most of the models analyzed were trained in contexts with good digital infrastructure and wide data availability.^31^, ^34^ These gaps in the literature suggest the need for prospective, multicenter studies with greater population diversity that consider social, ethnic‐racial, territorial, and structural factors that influence obstetric care. In addition, future research should incorporate mixed methods, combining quantitative approaches with qualitative studies, to understand not only the technical effectiveness of algorithms, but also their acceptance by health professionals and users, implementation barriers, and impacts on care processes.^26^, ^27^

Despite these limitations, the present study offers significant contributions to understanding the potential and challenges of using AI in maternal health care. By synthesizing recent scientific evidence, the review provides an updated overview of clinical applications of AI in risk prediction, clinical decision support, and monitoring of pregnant women across various contexts. Moreover, it highlights critical gaps such as the need to validate algorithms in diverse populations, invest in professional training, and develop public policies that address ethical, social, and structural dimensions. Therefore, this study represents a valuable contribution to guiding the design of safer, more effective, and more equitable strategies in reproductive and obstetric health.

## 5 CONCLUSIONS

This study reinforces that AI represents a promising innovation in gynecologic and obstetric health, with significant potential to reduce maternal morbidity and mortality, especially in contexts marked by structural inequalities and resource limitations. The evidence analyzed demonstrates that AI‐based tools, such as ML algorithms, neural networks and clinical decision support systems, have been effective in the early prediction of gestational complications, in supporting clinical decision making and in the continuous monitoring of pregnant and postpartum women.

From a political and institutional perspective, it is recommended that governments and health systems, particularly in low and middle‐income countries, invest in the local validation of AI algorithms, including sociodemographic and contextual variables that reflect the specificities of the populations served. This requires strengthening regional, interoperable and representative databases, as well as financing multicenter studies that evaluate the effectiveness and safety of these systems in scenarios of greater social vulnerability. In addition, public policies should provide mechanisms for regulation, ethical evaluation and continuous quality control of these technologies, with a view to preventing algorithmic biases and inequalities in digital access.

In the field of research, future investigations should prioritize the clinical impact of AI tools in real‐world care settings, considering both biomedical outcomes and the social, emotional, and cultural dimensions of technology use. Qualitative studies with health professionals and service users can help understand barriers and facilitators to AI adoption, contributing to the design of more context‐sensitive solutions. Regarding the humanistic dimension of care, it is essential that AI be conceived as a support and not as a substitute for clinical practice. The use of these technologies should strengthen clinical judgment, empathy and the bond between professionals and patients and not reduce them to automated or depersonalized interactions. To this end, it is essential to invest in the ethical and humanized training of professionals, including digital skills that favor the critical and reflective adoption of AI in obstetric care. In short, the integration of AI into maternal health must be aligned with the principles of social justice, reproductive rights and the humanization of care. Only by combining technological innovation, ethical commitment and social sensitivity will it be possible to transform AI into an effective tool in the fight against preventable maternal deaths.

## AUTHOR CONTRIBUTIONS

Gustavo Gonçalves dos Santos, substantial contributions to the conception or design of the work; or the acquisition, analysis, or interpretation of data for the work; preparing the work or revising it critically for important intellectual content; and final approval of the version to be published.

## FUNDING INFORMATION

None.

## CONFLICT OF INTEREST STATEMENT

The author has no conflicts of interest.

## Data Availability

Data sharing is not applicable to this article as no new data were created or analyzed in this study.
